# Association between occupational noise exposure and metabolic dysfunction-associated fatty liver disease: the mediating role of body mass index in automotive manufacturing workers

**DOI:** 10.3389/fpubh.2026.1793888

**Published:** 2026-07-07

**Authors:** Zhaoqian Chen, Jiaheng Yu, Sijia Ou, Yanmei Ruan, Xing Rong, Yuxia Zhang, Haijuan Huang, Jiaxin Cui, Zhi Wang

**Affiliations:** 1 Key Laboratory of Occupational Environment and Health, Guangzhou Twelfth People's Hospital, Guangzhou, China; 2 School of Public Health, Sun Yat-sen University, Guangzhou, China; 3 School of Public Health, North Sichuan Medical College, Nanchong, Sichuan, China

**Keywords:** metabolic dysfunction-associated fatty liver disease, occupational noise exposure, cumulative noise exposure, individual noise level, BMI

## Abstract

**Background:**

Occupational noise exposure has been linked to various adverse health outcomes, including metabolic disorders. However, limited evidence exists regarding its association with metabolic dysfunction-associated fatty liver disease (MAFLD). This study aimed to investigate the association between occupational noise exposure and MAFLD among automotive manufacturing workers, providing scientific evidence for the prevention and management of MAFLD in occupational populations.

**Methods:**

A cross-sectional study involving 3,427 male workers from two automobile manufacturing enterprises in Guangzhou, China, was conducted in 2023. Individual occupational noise exposure levels were assessed via cumulative noise exposure (CNE). MAFLD was diagnosed on the basis of ultrasound imaging and metabolic criteria, including obesity, type 2 diabetes, or metabolic dysregulation. Logistic regression models were used to evaluate the associations between CNE and MAFLD, adjusting for demographic, lifestyle, and clinical covariates. The interaction effects of shift work and hearing protection device use were analyzed. Mediation analysis was used to assess the role of body mass index (BMI) in the relationship between CNE and MAFLD.

**Results:**

According to the continuous models, each 1 dB-year increase in the CNE was associated with a 1.05-fold increased risk of MAFLD (OR: 1.05, 95% CI: 1.02, 1.08). According to the categorical models, workers in the highest CNE quartile (>89.651 dB-years) had a significantly greater risk of MAFLD (OR: 1.50, 95% CI: 1.05, 2.16) than did those in the lowest quartile. Subgroup analyses revealed that the association was more pronounced among workers who did not use hearing protection devices or worked night shifts (*p* for interaction<0.01). Body mass index (BMI) partially mediated the association between CNE and MAFLD, accounting for 3.48% of the total effect.

**Conclusion:**

Occupational noise exposure was associated with higher odds of MAFLD, and BMI partially mediated this association. Night/shift work and hearing protection use affect this relationship. These findings emphasize the need for effective noise control and occupational health interventions to mitigate MAFLD risk among workers exposed to noise.

## 1 Introduction

Metabolic dysfunction-associated fatty liver disease (MAFLD) is a chronic liver disorder strongly associated with metabolic dysfunction. With a global prevalence of approximately 25% (1), it has become the most common chronic liver disease worldwide, especially in populations with high rates of obesity and type 2 diabetes (2, 3). In addition to hepatic complications such as fibrosis, cirrhosis, and hepatocellular carcinoma, MAFLD substantially increases the risk of cardiovascular disease and all-cause mortality (4–7), posing a growing global public health concern (8). The development of MAFLD is multifactorial and involves genetic predispositions, dietary patterns, and environmental influences (9). While its pathophysiology remains incompletely understood, key mechanisms include insulin resistance, chronic inflammation, and oxidative stress (10). Emerging evidence further implicates occupational and environmental exposures, including noise pollution, as potential risk factors for MAFLD development (11), highlighting the need for further investigations in working populations.

High levels of occupational noise represent one of the most widespread workplace hazards today. A meta-analysis involving 108,256 participants revealed that the overall prevalence of occupational noise exposure in the general workforce is as high as 17% (12). In addition to its well-established effects on hearing, including tinnitus and hearing loss (13), noise exposure has been linked to sleep disturbances (14), increased irritability (15) and physiological stress responses mediated through activation of the hypothalamic–pituitary–adrenal (HPA) axis and the sympathetic nervous system (16). Emerging evidence suggests that noise may also affect liver metabolic health. Prolonged exposure to high-intensity noise may lead to elevated cortisol and adrenaline levels via chronic activation of the HPA axis and sympathetic pathways (11, 17). These hormonal responses are associated with insulin resistance and lipid metabolism dysfunction (18), which may contribute to hepatic fat accumulation and the onset and progression of MAFLD. While these findings point to a possible link between noise exposure and liver metabolic disorders, the underlying mechanisms remain to be fully elucidated.

Current research on the association between occupational noise and MAFLD is still in its early stages. A few epidemiological studies have suggested that workers with long-term noise exposure have increased rates of lipid metabolism disorders, metabolic syndrome, and obesity—established risk factors for MAFLD (19–21). However, direct evidence linking occupational noise to MAFLD is scarce, and most existing studies have focused on cardiovascular and general metabolic outcomes, with limited exploration of liver-specific effects (22). Furthermore, several methodological limitations in the current literature warrant attention. First, regarding disease definition, all four previous studies adopted traditional nonalcoholic fatty liver disease (NAFLD) or general fatty liver diagnostic criteria (ultrasound-detected hepatic steatosis) without incorporating core metabolic abnormalities (obesity and insulin resistance) (23–26). This may obscure potential interactions between noise exposure and metabolic dysfunction. Second, in terms of noise exposure assessment, with the exception of one study (26), the other three relied solely on occupational categories (manufacturing workers) as a proxy for noise exposure and lacked individualized quantitative data (decibel levels, daily exposure duration, cumulative exposure levels). Therefore, conducting high-quality prospective studies and mechanistic explorations is crucial to clarify the association between occupational noise and MAFLD and to elucidate its underlying mechanisms.

Using cross-sectional survey data from occupational populations in Guangzhou, southern China, we investigated the association between workplace noise exposure and metabolic dysfunction-associated fatty liver disease (MAFLD). We further assessed whether lifestyle behaviors and job-related characteristics modified this association. Finally, we conducted mediation analyses to explore whether body mass index (BMI) mediated the relationship between occupational noise exposure and MAFLD risk.

## 2 Methods

### 2.1 Study population

We conducted a cross-sectional study of automobile manufacturing workers in two automobile machining enterprises in Guangzhou, China, in 2023. The worker roster was obtained from the corporate occupational health department, covering main workshops, including stamping, welding, painting, and assembly, as well as supporting departments, with typical positions such as welders, casters, and assemblers. For further details on the workshops, workers’ roles, organizational structure, employee counts, and noise levels, please refer to our previously published work (27). The inclusion criteria were as follows: (1) had a duration of employment of ≥ 1 year; (2) were male workers; and (3) provided informed consent. The exclusion criteria were as follows: (1) lack of data on exposure or outcomes; and (2) severe cardiovascular and cerebrovascular diseases, severe hepatic and renal failure, malignancies, or other chronic cachectic conditions. Because women constitute only 1% of the workshop workers in this industry, with the majority of female employees serving as office clerks, female workers were excluded from the study to ensure sample representativeness and to account for noise exposure levels.

This investigation was conducted in strict accordance with the Declaration of Helsinki and was approved by the Ethics Review Committee of the Twelfth People’s Hospital of Guangzhou (Certificate Number: 2023055). Informed consent was obtained from all participants.

### 2.2 Definition of MAFLD

Fatty liver disease was diagnosed via abdominal ultrasonography performed by two experienced radiologists/sonographers. Hepatic steatosis, including increased liver echogenicity, vascular blurring, and deep attenuation was assessed according to standardized ultrasound criteria. MAFLD is defined on the basis of hepatic steatosis (28, 29) and any of the three elements listed below: obesity, type 2 diabetes, or metabolic abnormalities (8). Metabolic abnormalities were diagnosed if at least two of the following criteria were met:

(1) Blood pressure: systolic blood pressure (SBP) ≥ 130 mmHg, diastolic blood pressure (DBP) ≥ 85 mmHg or current use of antihypertensive medication.
(2) Triglyceride (TG): ≥1.7 mmol/L or current use of lipid-lowering medication.
(3) High-density lipoprotein cholesterol (HDL-C): Men <1.0 mmol/L, women <1.3 mmol/L or current use of lipid-regulating medication.
(4) Prediabetes: fasting plasma glucose (FPG) 5.6–6.9 mmol/L.

The severity of MAFLD was categorized into light MAFLD and moderate or heavy MAFLD. The specific classification criteria were as follows:

Light MAFLD: ultrasound findings indicated mild hepatic fat infiltration, characterized by a slight increase in liver parenchymal echogenicity, while the boundaries of the intrahepatic vessels and the diaphragm remained clearly visible. Moderate or heavy MAFLD: ultrasound findings indicate moderate-to-severe hepatic fat infiltration, characterized by a significant increase in liver parenchymal echogenicity, with blurred or invisible boundaries of intrahepatic vessels and the diaphragm.

### 2.3 Occupational noise assessment

Occupational noise exposure was assessed via the equivalent continuous A-weighted sound pressure level (LAeq), in accordance with China’s occupational standards (GBZ/T 229.4-2012). Noise levels were measured via a sound pressure meter (KSL TECHNOLOGY, KSL-dB2) following workplace measurement protocols outlined in GBZ/T 189.8–2007.

We employed tailored noise exposure assessment strategies on the basis of job characteristics. For roles with fluctuating noise levels (e.g., logistics and spot welding), personal noise dosimeters were used for full-shift monitoring. For stationary positions with relatively stable noise conditions, fixed-point measurements were used and matched to actual job assignments. Repeated measurements (≥3 per station across two shifts) were conducted to improve exposure representativeness. For stationary posts, the sound level meter was placed at the regular operating position near the worker’s breathing zone, without interfering with routine work, in accordance with the national occupational sampling standards.

To account for the intensity and duration of noise exposure, we quantified each participant’s exposure via cumulative noise exposure (CNE), calculated as noise level (dBA) × exposure duration (years), following the national standard (GBZ/T 189.8-2007), using the following formula:


CNE=10log[∑(10dB(A)10×T)]

### 2.4 Covariates

Demographic characteristics (age) and clinical conditions (hypertension, diabetes) were ascertained through medical examination records. Lifestyle factor (smoking, alcohol consumption, and physical activity) and sociodemographic information (education level, marital status, monthly income, and hearing protection use) were collected via a specifically designed questionnaire. Employment duration, weekly working days, and shift work data were extracted from factory archives. Smoking was defined as consuming ≥1 cigarette daily for ≥6 months; alcohol consumption as drinking ≥1 time weekly for ≥1 year; physical activity as ≥150 min of moderate-intensity exercise or ≥75 min of vigorous-intensity exercise per week; education was divided into junior high school or below and senior high school or above; marital status was categorized into three groups: ≤3,000, 3,000–8,000, and ≥8,000; use of hearing protection devices was recorded as “yes” or “no,” with “yes” indicating regular or almost daily use; and shift work and night work were binary variables, with “yes” indicating involvement in two-shift or three-shift rotations and working hours between 0:00 a.m. and 5:00 a.m.

### 2.5 Statistical analyses

The distribution of data was described across demographic, socioeconomic, behavioral, outcome, and exposure-related variables. The normality of the continuous data was evaluated via Q–Q plots and the Shapiro–Wilk test. Variables following a normal distribution are summarized as the means with standard deviations (SD), whereas those not normally distributed are presented as medians with interquartile ranges (IQR). Categorical variables are reported as frequencies and percentages. For group comparisons, analysis of variance (ANOVA) was applied to normally distributed continuous variables, followed by *t* tests for pairwise analyses. Nonnormally distributed continuous variables were analyzed via nonparametric rank-sum tests, and chi-square tests were used to assess differences in categorical variables among exposure groups.

Logistic regression models estimated the relationships as odds ratios (OR) with 95% confidence intervals (CI). Three models were fitted: an unadjusted model and two adjusted models incorporating covariates. Model 1 was adjusted for age, BMI, education, marital status, income, smoking status, alcohol consumption, physical activity, vegetable intake, meat intake, work days per week, shift work, night work, and hearing protection devices. Model 2 was further adjusted for hypertension, diabetes, total cholesterol (TC), triglycerides (TG) and high-density lipoprotein (HDL).

Additionally, potential effect modifiers, including smoking (yes vs. no), alcohol consumption (yes vs. no), physical activity (yes vs. no), shift work (shift vs. non-shift), and night shift (night shift vs. non-night shift), were tested. To facilitate the interpretation of interaction effects, the variables tested as effect modifiers were dichotomized. To assess the correlations among variables, Pearson’s correlation coefficients and point-biserial correlation coefficients were calculated for CNE, BMI, and MAFLD. Furthermore, to evaluate whether BMI mediated the association between cumulative noise exposure (CNE) and MAFLD, we performed mediation analysis with BMI specified as the mediator. The mediator model included BMI as the dependent variable, CNE as the independent variable, and covariates including age, education, marital status, income, smoking status, alcohol consumption, physical activity, vegetable intake, meat intake, work days per week, shift work, night work, hearing protection device use, hypertension, diabetes, total cholesterol, triglycerides, and high-density lipoprotein cholesterol. The outcome model included MAFLD status as the dependent variable, CNE as the exposure variable, BMI as the mediator, and the same covariates. The total effect, direct effect, indirect effect, and proportion mediated were estimated, and 95% confidence intervals were obtained using bootstrap resampling. Additionally, multicollinearity was assessed via the variance inflation factor (VIF) and tolerance values.

Statistical analyses were performed via R (version 4.1.2), with a two-sided significance level of *α* = 0.05.

## 3 Results

### 3.1 Basic demographic characteristics

A total of 3,427 automotive manufacturing workers were included in the analysis, 908 (26.5%) of whom were diagnosed with MAFLD. Table 1 presents the general characteristics of the study population. The median age was 28.0 years (IQR: 8.0), and the median BMI was 22.1 kg/m^2^ (IQR: 4.7). Significant differences in marital status, income, vegetable intake, meat intake, and the prevalence of hypertension and diabetes were detected between the non-MAFLD group and the MAFLD group. Compared with the non-MAFLD group, the MAFLD group presented significantly greater levels of CNE (*p* < 0.001), age (*p* < 0.001), BMI (*p* < 0.001), and length of service (*p* < 0.001). Additionally, within the MAFLD group, a greater proportion of individuals were married (*p* < 0.001), had higher income levels (*p* < 0.001), and reported greater daily intake of vegetables and meat (*p* < 0.001) than did the non-MAFLD group.

**Table 1:** General characteristics of the study participants (*N* = 3,427).

Characteristic	Total (*N* = 3,427)	Non-MAFLD (*N* = 2,519)	MAFLD (*N* = 908)	*p* value
Age^a^, years	28.0 (8.0)	27.0 (7.0)	32.0 (8.0)	<0.001
BMI^a^, kg/m^2^	22.1 (4.7)	21.0 (3.3)	25.2 (3.3)	<0.001
Smoking^b^				0.12
Yes	1,688 (49)	1,261 (50)	427 (47)	
Alcohol consumption^b^				0.4
Yes	1,262 (37)	918 (36)	344 (38)	
Physical activity^b^				0.9
Yes	655 (19)	483 (19)	172 (19)	
Education^b^				0.6
Senior high school and above	1,355 (40)	990 (39)	365 (40)	
Marital status^b^				<0.001
Single	1,776 (52)	1,506 (59.8)	270 (29.7)	
Married	1,604 (47)	989 (39.2)	615 (67.8)	
Others	47 (1.4)	24 (1.0)	23 (2.5)	
Income^b^, Yuan/month				<0.001
≤3,000	211 (6.2)	172 (6.8)	39 (4.3)	
3,001–8,000	2,569 (75)	1992 (79.1)	577 (63.5)	
≥8,000	647 (19)	355 (14.1)	292 (32.2)	
Vegetable intake^b^, g/day				<0.001
<300	1,572 (46)	1,187 (47.1)	385 (42.4)	
300–500	1,684 (49)	1,224 (48.6)	460 (50.7)	
>500	171 (5.0)	108 (4.3)	63 (6.9)	
Meat intake^b^, g/day				<0.001
<50	784 (23)	606 (24.1)	178 (19.6)	
50–75	2,168 (63)	1,594 (63.3)	574 (63.2)	
>75	475 (14)	319 (12.6)	156 (17.2)	
Noise^a^, dB(A)	78.5 (4.0)	78.5 (3.9)	78.5 (4.3)	0.8
CNE^a^, dB(A)-year	86.0 (7.6)	85.3 (7.4)	88.6 (6.4)	<0.001
Length of service^a^, years	5.7 (9.7)	4.8 (7.2)	11.7 (10.2)	<0.001
Work days per week^a^, days	5.0(1.0)	5.0(1.0)	5.0(1.0)	0.698
Shift work^b^				<0.001
Yes	3,066 (89)	2,289 (91)	777 (86)	
Night work^b^				<0.001
Yes	3,010 (88)	2,255 (90)	755 (83)	
Hearing protection devices^b^				0.007
Yes	2,352 (69)	1,751 (70)	601 (66)	
Hypertension^b^				<0.001
Yes	116 (3.4)	52 (2.1)	64 (7.0)	
Diabetes^b^				<0.001
Yes	36 (1.1)	7 (0.3)	29 (3.2)	
TC^a^	4.9 (1.2)	4.8 (1.2)	5.2 (1.2)	<0.001
TG^a^	1.1 (0.9)	1.0 (0.7)	1.7 (1.3)	<0.001
HDL^a^	1.4 (1.4)	1.4 (0.4)	1.2 (0.3)	<0.001

BMI, body mass index; CNE, cumulative occupational noise exposure; IQR, interquartile range; TC, total cholesterol; TG, triglyceride; HDL, high-density lipoprotein.

a Median (IQR) is used to describe this variable.

b *N* (%) is used to describe this variable; *p* value reflects the difference between the two groups (normal weight, overweight/obesity).

### 3.2 Correlation between occupational noise exposure and MAFLD

Table 2 presents the associations between cumulative noise exposure (CNE) and MAFLD. In the crude model, each 1 dB(A)-year increase in CNE was linked to an 11.5% higher odds of overall MAFLD (OR = 1.115, 95% CI: 1.097:1.134), with the highest CNE quartile (Q4: >89.651 dB(A)-years) showing a 4.53-fold higher odds (95% CI: 3.60:5.74) compared with the lowest quartile (Q1). These associations remained significant, although attenuated, after adjusting for sociodemographic, lifestyle, and metabolic factors (adjusted Model 2: continuous OR = 1.023, 95% CI: 0.998:1.049; Q4 OR = 1.50, 95% CI: 1.05:2.16). A similar pattern was observed for light MAFLD, where continuous CNE initially increased the risk by 11.0% per dB(A)-year (OR = 1.109, 95% CI: 1.090:1.128), with Q4 exhibiting a 4.18-fold greater risk (95% CI: 3.29:5.34); this association persisted after adjustment (adjusted Model 2: Q4 OR = 1.51, 95% CI: 1.05:2.19). For moderate-to-heavy MAFLD, only quartile-based analysis revealed significant associations (adjusted model 2: Q4 OR = 2.73, 95% CI: 1.01: 8.09).

**Table 2:** Associations between cumulative noise exposure and MAFLD (*N* = 3,427).

Subgroup	CNE (continuous)^a^ dB(A)-years	CNE (IQR)				
		Q1 (≤82.032) dB(A)-years	Q2 (82.032–86.039) dB(A)-years	Q3 (86.039–89.651) dB(A)-years	Q4 (>89.651) dB(A)-years	*p* for trend
	OR (95%CI)	OR (95%CI)	OR (95%CI)	OR (95%CI)	OR (95%CI)	
MAFLD						
Crude model	**1.115 (1.097, 1.134)**	Reference	**1.503 (1.169, 1.936)**	**2.203 (1.733, 2.812)**	**4.534 (3.602, 5.739)**	**<0.001**
Adjusted Model 1^b^	**1.026 (1.002, 1.051)**	Reference	1.374 (0.993, 1.907)	**1.446 (1.048, 2.001)**	**1.526 (1.076, 2.171)**	**0.026**
Adjusted Model 2^b^	1.023 (0.998, 1.049)	Reference	1.287 (0.923, 1.798)	**1.396 (1.002, 1.950)**	**1.503 (1.050, 2.157)**	**0.030**
Light-MAFLD						
Crude model	**1.109 (1.090, 1.128)**	Reference	**1.397 (1.074, 1.821)**	**2.110 (1.645, 2.718)**	**4.181 (3.293, 5.339)**	**<0.001**
Adjusted Model 1^b^	**1.025 (1.000, 1.051)**	Reference	1.379 (0.991, 1.924)	**1.454 (1.049, 2.024)**	**1.534 (1.073, 2.199)**	**0.027**
Adjusted Model 2^b^	1.022 (0.997, 1.048)	Reference	1.289 (0.919, 1.811)	**1.420 (1.015, 1.994)**	**1.513 (1.050, 2.187)**	**0.029**
Moderate or heavy-MAFLD						
Crude model	**1.158 (1.113, 1.208)**	Reference	**2.862 (1.367, 6.547)**	**3.400 (1.640, 7.731)**	**9.093 (4.677, 19.877)**	**<0.001**
Adjusted Model 1^b^	1.053 (0.990, 1.121)	Reference	**3.141 (1.210, 9.055)**	**2.673 (1.018, 7.781)**	**2.869 (1.080, 8.424)**	0.161
Adjusted Model 2^b^	1.051 (0.985, 1.122)	Reference	**3.174 (1.219, 9.169)**	2.144 (0.796, 6.311)	**2.731 (1.010, 8.092)**	0.224

CNE, cumulative noise exposure; IQR, interquartile range; OR, odds ratio.

a Per 10 dB-year change in the CNE level.

b VIF < 5 and tolerance >0.1 for all factors.

The significance of bold is *p* < 0.05.

Model 1 was adjusted for age, BMI, education, marital status, income, smoking status, alcohol consumption, physical activity, vegetable intake, meat intake, work days per week, shift work, night work, and hearing protection devices.

Model 2: Adjusted Model 1 + hypertension, diabetes, TC, TG, and HDL.

### 3.3 Interaction analysis

**Figure 1:**
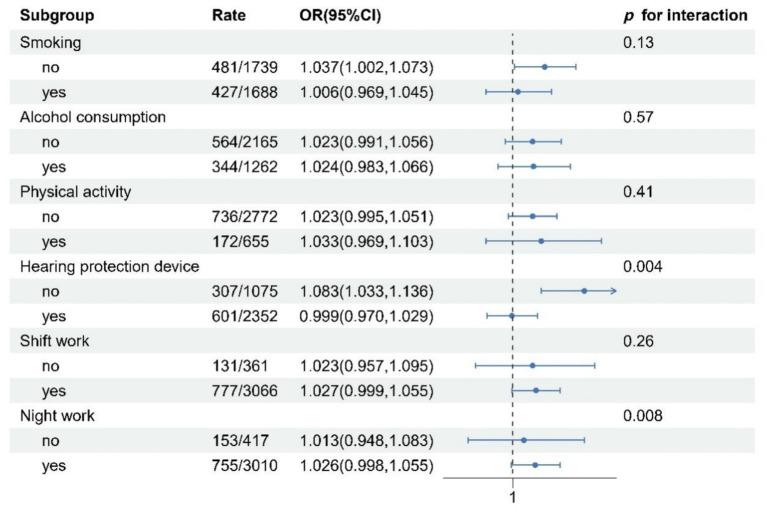
Interaction analysis of associations between cumulative noise exposure and MAFLD among different subgroups. Adjusted for age, BMI, education, marital status, income, smoking, alcohol consumption, physical activity, vegetable intake, meat intake, work days per week, shift work, night work, hearing protection devices, hypertension, diabetes, TC, TG, and HDL.

**Figure 2:**
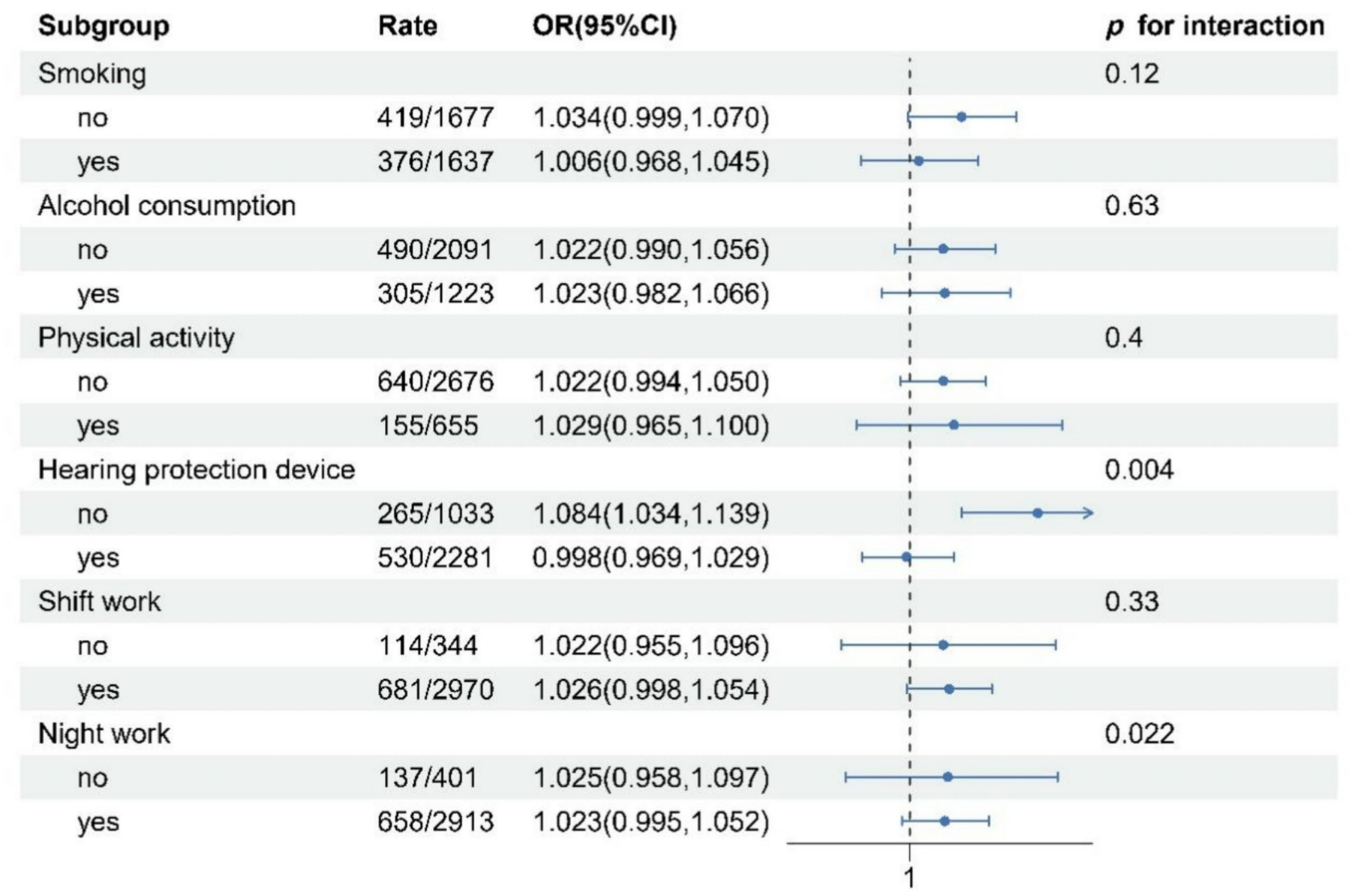
Interaction analysis of associations between cumulative noise exposure and Light-MAFLD among different subgroups. Adjusted for age, BMI, education, marital status, income, smoking, alcohol consumption, physical activity, vegetable intake, meat intake, work days per week, shift work, night work, hearing protection devices, hypertension, diabetes, TC, TG, and HDL.

**Figure 3:**
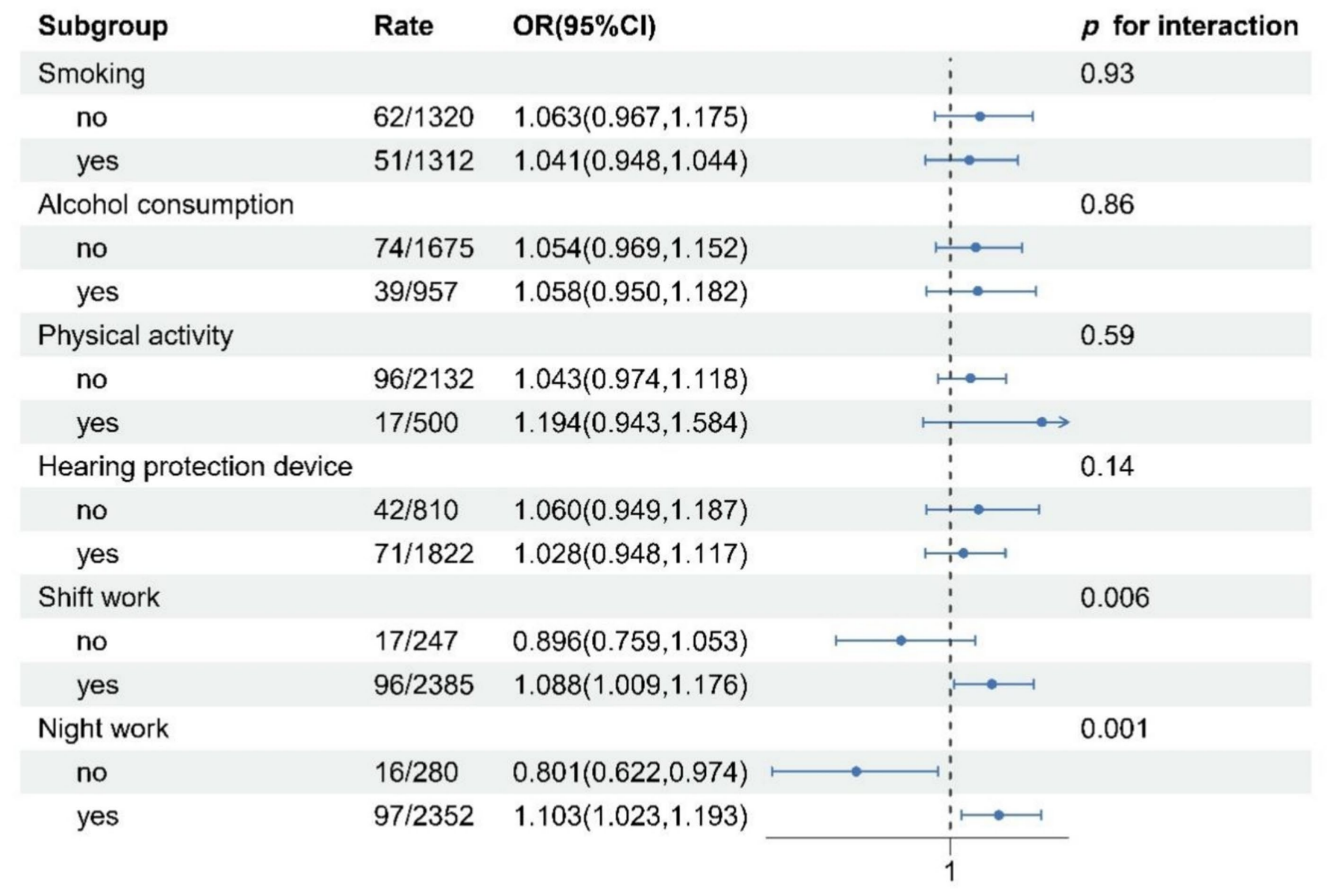
Interaction analysis of associations between cumulative noise exposure and moderate or heavy-MAFLD among different subgroups. Adjusted for age, BMI, education, marital status, income, smoking, alcohol consumption, physical activity, vegetable intake, meat intake, work days per week, shift work, night work, hearing protection devices, hypertension, diabetes, TC, TG, and HDL.

Figures 1–3 present the interaction analysis of the associations between CNE and MAFLD among different subgroups. For MAFLD, in the population of workers who do not use hearing protection devices or need to work night shifts, the association between occupational noise exposure and MAFLD is more pronounced (*p*_for interaction_ = 0.004 for hearing protection devices, *p*_for interaction_ = 0.008 for night work). Conversely, we did not observe significant effects of smoking, alcohol consumption, lack of physical exercise, or shift work. Similar trends were observed for Light-MAFLD. The association between occupational noise exposure and moderate or heavy MAFLD was significantly more pronounced among shift workers and night workers (*p*_for interaction_ = 0.006 for shift work; *p*_for interaction_ = 0.001 for night work).

### 3.4 Analysis of BMI mediation

**Figure 4:**
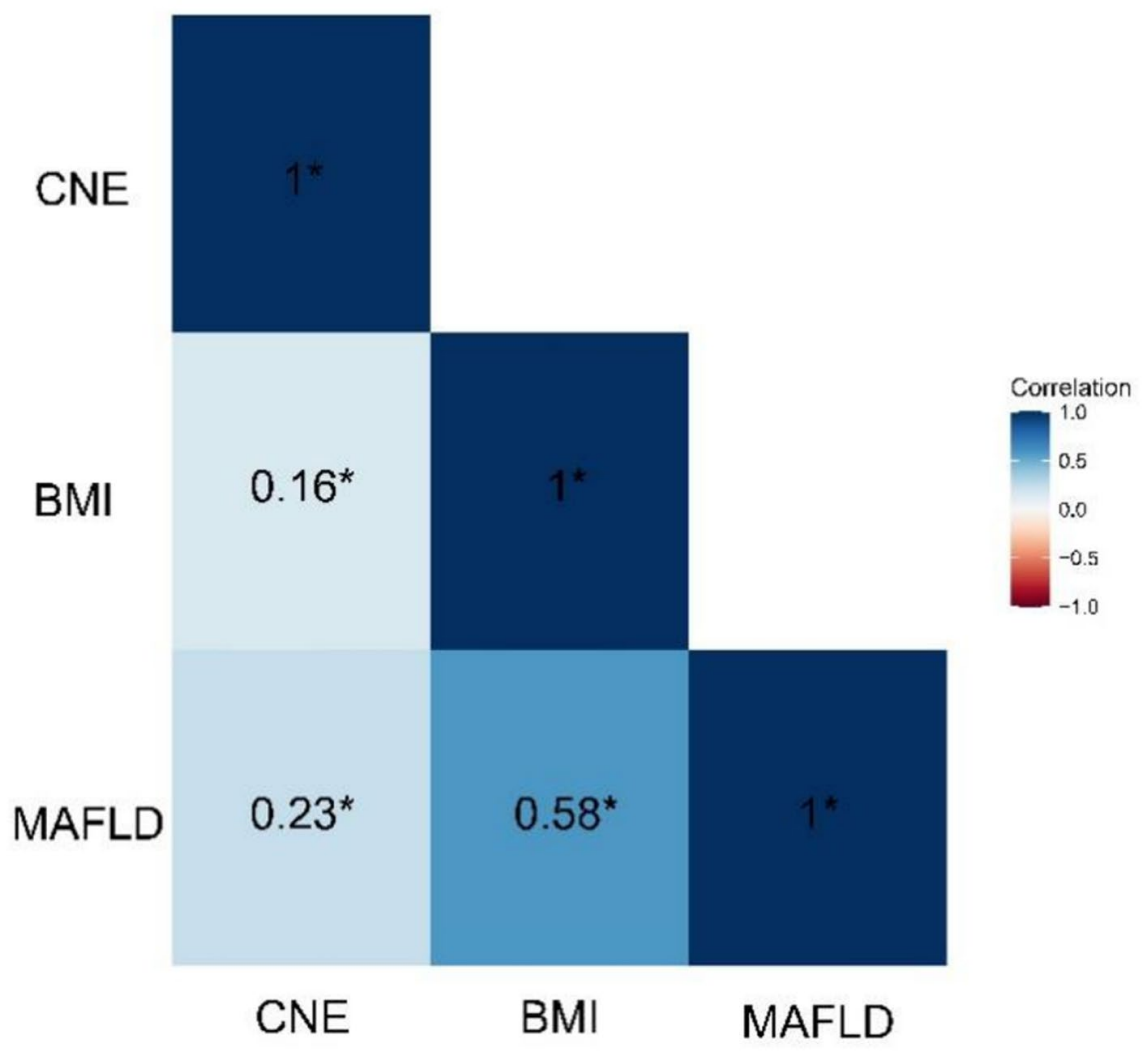
Pearson’s coefficient and point-biserial correlation coefficient for CNE, BMI, and MAFLD. ^*^p < 0.05.

The results of the Pearson and point-biserial correlation analyses, as illustrated in Figure 4, revealed three statistically significant associations (all *p* < 0.05): (1) a significant positive correlation between CNE and BMI (*r* = 0.16); (2) a significant association between CNE and MAFLD status (*r* = 0.23); and (3) a significant correlation between BMI and MAFLD (*r* = 0.58). These observed correlations were compatible with the basic statistical conditions for conducting exploratory mediation analysis. However, correlation alone does not establish a causal mediation pathway, and the mediation findings should be interpreted cautiously.

The possible effects of BMI on the association of CNE with MAFLD are shown in Table 3. These findings indicated that BMI mediated 3.48 and 3.3% of the associations of CNE with MAFLD and Light-MAFLD, respectively. However, for moderate or heavy MAFLD, the mediating effect of BMI was not significant.

**Table 3:** Mediation analysis of BMI in the link between cumulative noise exposure and MAFLD (*N* = 3,427).

Subgroup	Total effect (95%CI)	Direct effect (95%CI)	Indirect effect (95%CI)	Proportion mediated (%)
BMI				
MAFLD	**0.0004 (0.0001–0.0006)**	**0.0003 (0.0001, 0.0005)**	**0.0001 (0.0000, 0.0001)**	**3.48%**
Light-MAFLD	**0.0003 (0.0000, 0.0005)**	0.0002 (−0.0000, 0.0005)	**0.0001 (0.0000, 0.0001)**	3.3%
Moderate or heavy-MAFLD	−0.0000 (−0.0001, 0.0001)	−0.0000 (−0.0001, 0.0001)	0.0000 (−0.0000, 0.0001)	2.1%

CNE, cumulative noise exposure; IQR, interquartile range; OR, odds ratio.

The significance of bold is *p* < 0.05.

Adjusted for age, BMI, education, marital status, income, smoking, alcohol consumption, physical activity, vegetable intake, meat intake, work days per week, shift work, night work, hearing protection devices, hypertension, diabetes, TC, TG, and HDL.

## 4 Discussion

Building on limited prior evidence, this study further investigated the association between individually assessed occupational noise exposure and MAFLD in a Chinese working population, providing additional insight into potential environmental contributors to metabolic liver disease. By assessing cumulative noise exposure (CNE) at the individual level, we provide new epidemiological evidence on the role of occupational noise in the development of MAFLD. Our findings revealed a significant positive association between CNE and MAFLD risk. Notably, the associations observed in the CNE quartile models appeared stronger than those in the continuous models. In particular, workers in the highest CNE quartile had higher odds of MAFLD than those in the lowest quartile, whereas the continuous association was relatively modest after multivariable adjustment. This pattern may suggest a possible non-linear exposure-response relationship or threshold effect, whereby adverse metabolic liver outcomes become more apparent after cumulative noise exposure exceeds a certain level. However, because the present study was cross-sectional and the categorical analysis may also reflect differences in exposure duration or other unmeasured occupational factors, this finding should be interpreted cautiously. Future studies using prospective designs and flexible modeling approaches, such as restricted cubic splines, are needed to further evaluate potential non-linear associations. Furthermore, the use of hearing protection devices and night shift work were identified as effect modifiers in the relationship between occupational noise exposure and MAFLD. However, because several subgroup and interaction analyses were conducted, some statistically significant interaction findings may have occurred by chance due to multiple comparisons. These results should therefore be interpreted as exploratory and require confirmation in future studies. Additionally, BMI partially mediated the relationship between CNE and MAFLD. From an occupational health perspective, our findings suggest that workplace noise prevention may have implications beyond hearing conservation. Regular assessment of cumulative noise exposure, engineering noise control, administrative measures to reduce exposure duration, and consistent use of hearing protection devices may be important components of workplace health protection. In addition, workers with high cumulative noise exposure, night work, or irregular shift schedules may benefit from targeted health surveillance, including monitoring of body weight, metabolic indicators, and liver ultrasound findings when clinically appropriate. These strategies may help identify high-risk workers earlier and support integrated prevention of both auditory and non-auditory health outcomes in noise-exposed occupational populations.

### 4.1 Summarizing existing evidence and our study’s contribution

Several studies have explored the relationship between occupational noise exposure and fatty liver disease. To date, only four cross-sectional studies have specifically examined this association in occupational populations (23–26), all reporting a positive correlation between noise exposure and the prevalence of fatty liver disease. For example, a study in Guangzhou, China, reported an elevated risk of fatty liver disease among workers exposed to both benzene and occupational noise (24). Another study in a confectionery manufacturing enterprise reported a higher prevalence of fatty liver disease in noise-exposed workers, particularly among males (25). In Tianjin, a study of 2,333 workers using pure-tone audiometry divided participants into normal hearing and hearing loss groups, revealing that the latter had significantly higher rates of hyperlipidemia and fatty liver disease (23). Zhang et al. (26) reported that the CNE may be an independent risk factor for NAFLD. Supporting evidence from animal models reinforces this link. Chronic noise exposure in mice fed a high-fat diet exacerbated fatty liver progression (11), whereas prolonged noise exposure initially reduced weight but later increased fat mass and adipocyte hypertrophy (30). These findings align with human epidemiological data, suggesting that long-term noise exposure may disrupt metabolic homeostasis through pathways such as stress response activation, sleep disturbances, and systemic inflammation (21, 31–33). After adjusting for multiple covariates, our study demonstrated a clear association between occupational noise exposure and MAFLD.

### 4.2 Heterogeneity of association across MAFLD severity

Our study also revealed that the association between CNE and MAFLD varied across different severity levels of MAFLD. The association was more pronounced in individuals with mild MAFLD, whereas it appeared attenuated in those with moderate-to-severe disease. One possible explanation is that individuals with mild MAFLD may be more susceptible to external environmental stressors such as noise. In contrast, among those with advanced disease, metabolic dysfunction may already be dominated by more profound pathophysiological changes, thereby diminishing the relative contribution of noise exposure (34).

### 4.3 Modifiable risk factors: shift work and hearing protection

Our study revealed statistically significant associations between night/shift work and the use of hearing protection devices with MAFLD occurrence. With respect to shift work, individuals who engaged in night or rotating shifts were more likely to have moderate-to-severe MAFLD. This observation aligns with prior large-scale studies. For example, a prospective cohort study involving over 280,000 participants revealed that longer shift durations, frequent night shifts, and extended working hours were associated with increased NAFLD risk (35). Another study among young adult workers also revealed a significant link between shift work and NAFLD development (36). One possible explanation is the disruption of circadian rhythms, which impairs melatonin and cortisol secretion (37), leading to insulin resistance and abnormal lipid metabolism. Additionally, shift workers often experience sleep disturbances and irregular eating patterns, such as frequent consumption of calorie-dense, high-fat foods (38, 39), which may further exacerbate metabolic dysregulation. In contrast, appropriate use of hearing protection devices was associated with a lower risk of MAFLD. By reducing the intensity of occupational noise exposure, HPD use may limit physiological stress responses and metabolic disruptions linked to chronic noise. Taken together, our findings highlight night/shift work and HPD use as modifiable factors influencing MAFLD risk in high-noise occupational environments. Targeted interventions addressing work schedules and noise protection could therefore contribute to disease prevention.

### 4.4 Potential pathway: partial mediation by body mass index

We further examined the mediating role of body mass index (BMI) in the association between cumulative noise exposure (CNE) and MAFLD. Mediation analysis demonstrated that BMI partially mediated this relationship, suggesting that occupational noise exposure is associated with higher odds of MAFLD, partly through its relationship with BMI. This finding is consistent with existing evidence linking noise exposure to metabolic disturbances via multiple biological pathways. First, chronic noise exposure may promote weight gain by activating the HPA axis, leading to elevated cortisol levels that disrupt appetite regulation and energy homeostasis (40). Second, experimental studies have indicated that noise-induced stress can directly alter lipid metabolism and adipose tissue function (41, 42). The mediating role of BMI in our study is biologically plausible given the well-established metabolic consequences of obesity. Elevated BMI impairs liver metabolic function by increasing free fatty acid release (43) and inducing insulin resistance (44). These obesity-related metabolic disturbances have been consistently associated with both the incidence and progression of MAFLD in population-based studies (45).

### 4.5 Exploring underlying pathophysiological mechanisms

Emerging evidence suggests multiple pathways through which noise exposure may contribute to the pathogenesis of MAFLD, although the precise mechanisms require further elucidation. Noise acts as a chronic stressor that activates the HPA axis, resulting in sustained elevation of cortisol levels (11, 18). This hormonal imbalance promotes visceral fat accumulation, particularly in the liver, thereby increasing intracellular lipid deposition and exacerbating hepatic steatosis (46). The development of insulin resistance further impairs hepatic lipid metabolism by increasing the influx of free fatty acids into hepatocytes, facilitating MAFLD progression (47). In addition to metabolic dysregulation, noise exposure has been linked to increased oxidative stress and inflammatory responses, which can damage hepatocytes and promote hepatic fat accumulation (48). Additionally, chronic noise-induced disruption of sleep quality and circadian rhythms may contribute to metabolic disturbances that increase MAFLD risk (49). Elevated liver enzymes, such as alanine aminotransferase (ALT) and aspartate aminotransferase (AST), observed among noise-exposed individuals provide further evidence of hepatic dysfunction and cellular injury in this context (50, 51). Epidemiological studies also indicate associations between occupational noise exposure and increased risks of hyperlipidemia and fatty liver disease (21). Collectively, these findings support that occupational noise exposure is associated with alterations in liver metabolic function through multiple, interconnected biological pathways. Nevertheless, additional research is needed to confirm causal relationships and clarify the underlying mechanisms involved.

### 4.6 Limitations

This study has several limitations that warrant consideration. First, its cross-sectional design precludes establishing a causal relationship between occupational noise exposure and MAFLD. Second, although our exposure assessment followed national occupational standards, some stationary posts were assessed by fixed-point short-duration measurements rather than full-shift personal monitoring, which may not have fully captured within-shift variability and may have introduced non-differential exposure misclassification. Third, the diagnosis of MAFLD did not include certain metabolic indicators, such as waist circumference, hemoglobin A1c, and plasma high-sensitivity C-reactive protein levels, which may have resulted in the misclassification of some participants. Fourth, alcohol consumption was collected as a dichotomous self-reported variable rather than a quantitative exposure metric; therefore, we could not evaluate dose–response relationships or perform finer stratified analyses, and residual confounding by alcohol intake cannot be excluded. Fifth, female workers were excluded because they accounted for only a very small proportion of workshop workers in the participating enterprises and were mainly employed in office-based positions with relatively low noise exposure. Therefore, our findings may not be generalizable to female workers, workers in other industries, or occupational populations with different noise exposure patterns. Further studies including women and more diverse occupational settings are warranted. Sixth, Although we adjusted for a range of demographic, lifestyle, occupational, and clinical covariates, residual confounding cannot be excluded. Some MAFLD-related factors, including overall dietary quality, total caloric intake, detailed medication use, family history of metabolic disorders or liver disease, and other occupational exposures such as chemical agents, heat, dust, or psychosocial stress, were not fully assessed in this study. These unmeasured or incompletely measured factors may have influenced the observed associations. Despite these limitations, this study has notable strengths. We employed individual-level noise exposure measurements, allowing for more refined exposure assessment than occupation-based proxies. Additionally, subgroup analyses facilitated the identification of high-risk populations, providing valuable insights for the development of targeted prevention strategies.

## 5 Conclusion

This study suggests that occupational noise exposure was associated with higher odds of MAFLD. These findings highlight the potential impact of workplace noise on metabolic health, emphasizing the need for effective noise control measures and regular health monitoring in occupational settings. Further research is warranted to explore the underlying mechanisms and long-term health implications of noise exposure.

## Data Availability

The data that support the findings of this study are not openly available due to reasons of sensitivity and are available from the corresponding author upon reasonable request. Data are located in controlled access data storage at Guangzhou Twelfth People’s Hospital.
